# Implementation of a novel computer assisted telephone follow-up model for older patients after emergency department discharge in an Asian population

**DOI:** 10.1007/s40520-024-02796-6

**Published:** 2024-07-18

**Authors:** Yen-Chiang Lee, Sam Yu-Chieh Ho, Tian-Hoe Tan, Chung-Han Ho, Kang-Ting Tsai, Pei-Chi Yang, Chien-Chin Hsu, Hung-Jung Lin, Chia-Ti Wang, Chien-Cheng Huang

**Affiliations:** 1 Department of Emergency Medicine, Chi Mei Medical Center, 901 Zhonghua Road, Yongkang District, Tainan City, 710 Taiwan; 2 Department of Senior Welfare and Services, Southern Taiwan University of Science and Technology, Tainan, Taiwan; 3 Department of Medical Research, Chi Mei Medical Center, Tainan, Taiwan; 4 Department of Information Management, Southern Taiwan University of Science and Technology, Tainan, Taiwan; 5 Division of Geriatrics and Gerontology, Department of Internal Medicine, Chi Mei Medical Center, Tainan, Taiwan; 6 School of Medicine, College of Medicine, National Sun Yat-sen university, Kaohsiung, Taiwan; 7 Department of Emergency Medicine, Taipei Medical University, Taipei, Taiwan; 8 Department of Emergency Medicine, Kaohsiung Medical University, Kaohsiung, Taiwan

**Keywords:** Computer, Discharge, Emergency department, Older, Outcome, Telephone follow-up

## Abstract

**Background:**

While the impact of telephone follow-up (TFU) for older emergency department (ED) patients is controversial, its effects on the Asian population remain uncertain. In this study, we evaluated the effectiveness of a novel computer assisted TFU model specifically for this demographic.

**Methods:**

At a Taiwanese tertiary medical center, we developed a TFU protocol that included a referral and case management system within the ED hospital information system. We provided TFU to older discharged patients between April 1, 2021, and May 31, 2021. We compared this cohort with a non-TFU cohort of older ED patients and analyzed demographic characteristics and post-ED discharge outcomes.

**Results:**

The TFU model was successfully implemented, with 395 patients receiving TFU and 191 without TFU. TFU patients (median age: 76 years, male proportion: 48.9%) differed from non-TFU patients (median age: 74 years, male proportion: 43.5%). Compared with the non-TFU cohort, the multivariate logistic regression analysis revealed that the TFU cohort had a lower total medical expenditure < 1 month (adjusted odds ratio [AOR]: 0.32; 95% CI: 0.21 − 0.47 for amounts exceeding 5,000 New Taiwan Dollars), and higher satisfaction (AOR: 2.80; 95% CI: 1.46 − 5.36 for scores > 3 on a five-point Likert Scale). However, the TFU cohort also had a higher risk of hospitalization < 1 month (AOR: 2.50; 95% CI: 1.31 − 4.77) compared to the non-TFU cohort.

**Conclusion:**

Computer-assisted TFU appears promising. Further research involving a larger number of patients and validation in other hospitals is necessary to bolster the evidence and extend the findings to a broader context.

**Supplementary Information:**

The online version contains supplementary material available at 10.1007/s40520-024-02796-6.

## Introduction

As the global population ages, addressing the challenges of aging becomes a critical public health issue. In 2020, the older population, defined as individuals older than 65 years old, accounted for 16.9% of the total population, and it is projected to increase to 19.0% by 2025 in the United States [1]. Similarly, Taiwan experienced a rise in the older population, reaching 16.2% in 2021, and it is expected to further escalate to 20% by 2025 [2]. Emergency departments (EDs) are the primary healthcare facilities where older individuals seek emergency care [3]. Most older ED patients are discharged after evaluation and treatment [4]. However, older adults discharged from the ED are at a higher risk of adverse outcomes compared to the younger population, including functional decline, ED return visits, hospitalization, and mortality [4, 5]. Several risk factors contribute to these outcomes, such as pre-existing functional and cognitive impairments, lack of social support, living alone, and experiencing depression [5]. Consequently, older patients discharged from the ED may require more transitional care or close medical surveillance due to their increased vulnerability to medical deterioration [4, 5].

Telephone follow-up (TFU) after ED discharge has been proposed as a method to improve patient compliance, clarify discharge instructions, and address any issues that may have arisen after leaving the ED [5]. However, the literature on the effect of TFU for older ED patients remains controversial [5]. A systematic review of two published randomized controlled studies revealed no significant benefits of TFU regarding ED return visits, hospitalization, acquisition of prescribed medication, and compliance with follow-up appointments in older ED patients [5–7]. Numerous factors may influence the outcomes of TFU in older ED patients, including the specific protocols adopted by the study hospital and variations in insurance, medical resources, and cultural factors. Notably, there is a lack of studies focusing on this issue in the Asian population. In response to these challenges, the Chi Mei Medical Center (CMMC) established a geriatric ED in 2019 to enhance the quality of care for older ED patients [8]. Various geriatric care protocols have been implemented and published, encompassing hospice and palliative care, geriatric syndrome screening, medication reconciliation, delirium screening, treatment, and referrals for home healthcare [9–14]. Within this context, we developed a novel computer-assisted model for TFU and conducted this study to elucidate its impact on the Asian population.

## Materials and methods

### Study design, setting, and participants

**Fig. 1:**
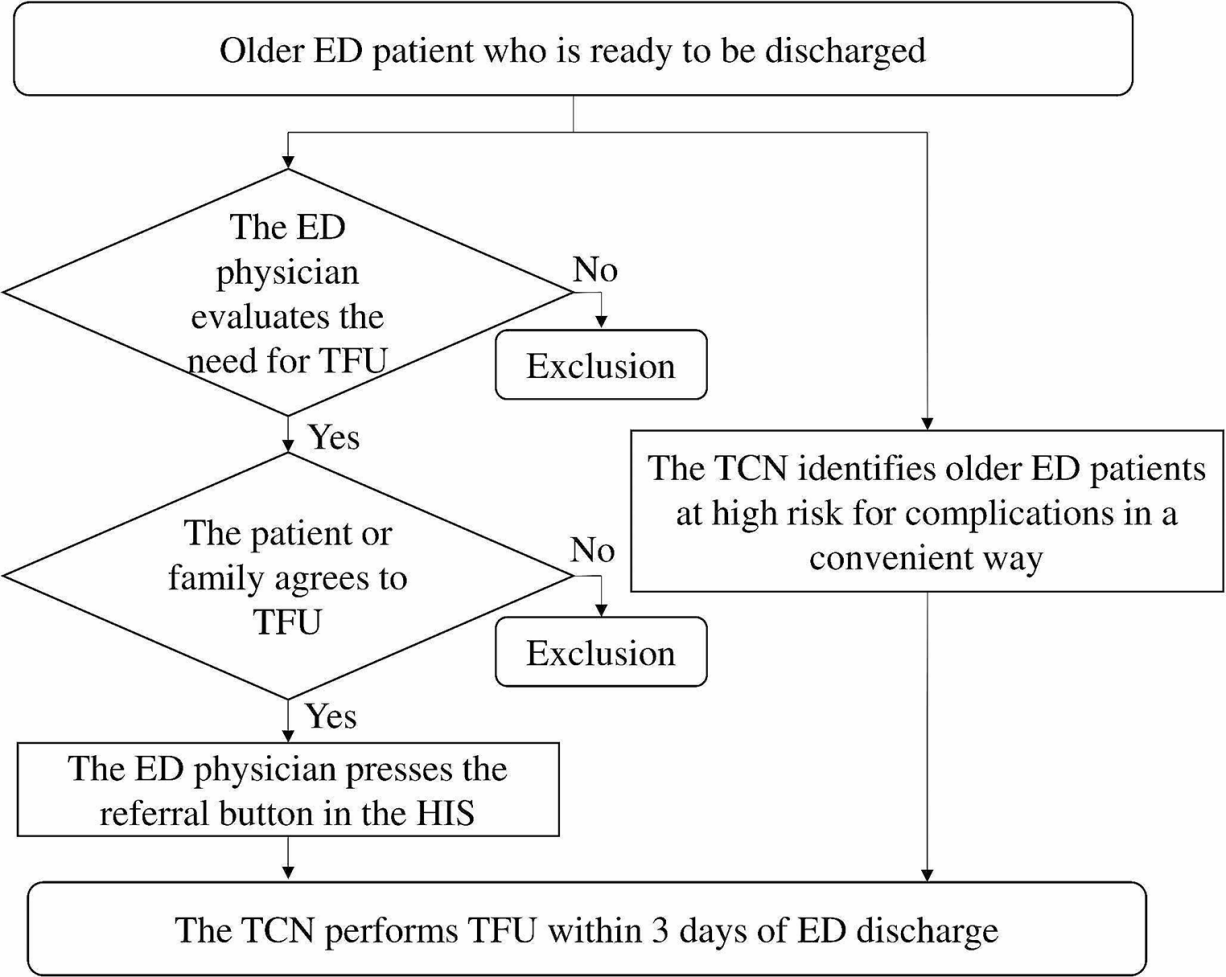
The flowchart illustrates the computer assisted TFU referral protocol. ED, emergency department; TFU, telephone follow-up; TCN, transitional care nurse; HIS, hospital information system

We conducted this study at CMMC, a tertiary medical center in Southern Taiwan. In 2021, the CMMC ED had around 60 full-time physicians and residents, attending to over 300 patients daily. The geriatric ED at CMMC comprises a team of interdisciplinary members, including ED physicians, dedicated transitional care nurse (TCN), nurse practitioners, nurses, geriatricians, social workers, pharmacists, physical therapists, and dietitians [8]. The dedicated TCN has 10 years of working experience in the ED and is well-trained in geriatric emergency care. The TCN’s work hours were from 08:00 AM to 05:00 PM, Monday to Friday. The interdisciplinary team maintains close collaboration, holding small group meetings every week and a general meeting every month. Firstly, we established a referral system in the hospital information system (HIS) of the ED, along with a case management system). The referral button in the HIS was designed to facilitate quick and straightforward referrals by ED physicians. The case management system allowed the TCN to oversee these patients, capturing essential data and follow-up outcomes. Secondly, we developed a referral protocol after discussions with the interdisciplinary team (Fig. 1). The inclusion criteria for patients were age ≥ 65 years-old and discharge from the ED. Two methods were used to select patients: (1) referrals from the ED physicians or (2) identification by the TCN of older ED patients at high risk for complications, using a convenient approach. The general criteria for selecting patients for TFU by ED physicians and the TCN involved a comprehensive assessment of the patient’s condition and social support. Dementia was not an absolute criterion for TFU. The more detailed criteria included: (1) high risk for disease progression; (2) discharge against medical advice; (3) poor social support at home; (4) significant distance from home to hospital, causing inconvenience for returning to the hospital; (5) any other issues that ED physicians and the TCN deemed necessary for TFU. The TCN performed telephone or video call follow-ups within 3 days of ED discharge.

### Data collection

**Fig. 2:**
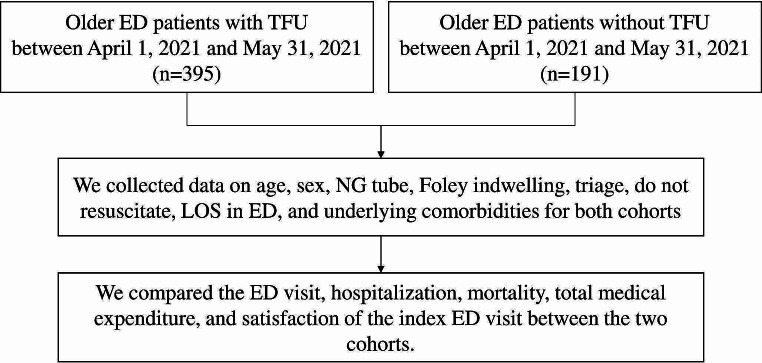
Inclusion of TFU and non-TFU cohorts for comparison analyses. TFU, telephone follow-up; ED, emergency department; NG, nasogastric; LOS, length of stay

We retrospectively collected data for the patients who underwent TFU between April 1, 2021, and July 31, 2021, forming the study cohort (Fig. 2). Simultaneously, we randomly selected the comparison cohort from older ED patients who were discharged but did not undergo TFU during the same period. The selection criteria for non-TFU patients were the same as for TFU patients; however, these patients or their caregivers refused TFU. The TCN nurse selected non-TFU patients using a convenient method. Data on age, sex, nasogastric tube, Foley indwelling, triage, do not resuscitate status, length of stay (LOS) in the ED, and underlying comorbidities were collected for both cohorts.

### Outcome measurements

We compared the rates of ED revisits < 3 days, ED revisits < 1 month, hospitalizations < 14 days, hospitalizations < 1 month, mortality < 1 month, total medical expenditure < 1 month, and satisfaction of the index ED visit (measured on a five-point Likert scale) between the two cohorts during a one-month follow-up period. The total medical expenditure was calculated based on the National Health Insurance reimbursements [15]. Satisfaction was measured using a five-point Likert scale, which includes the following options: (1) Strongly Disagree, (2) Disagree, (3) Neither Agree nor Disagree, (4) Agree, and (5) Strongly Agree [16]. In the TFU cohort, the TCN assessed satisfaction with the index ED visit and TFU service after engaging in discussion and education with the patient and caregiver. In the non-TFU cohort, the TCN assessed satisfaction with the index ED visit only.

### Ethical statements

We conducted this study with the approval of the institutional review board of CMMC, and we also anonymized all patient data. Since our study was retrospective and observational, patient informed consent was not required.

### Statistics

The Mann-Whitney U Test was employed for analyzing ordinal or continuous variables that were not normally distributed. The T-test, on the other hand, was used for analyzing ordinal or continuous variables with a normal distribution. For categorical variable analyses, we used either the Chi-square test or Fisher’s Exact Test. To assess the odds ratios of outcomes, total medical expenditure, and satisfaction in older ED patients with TFU compared to those without TFU, we performed logistic regression analyses. Statistical Analysis System 9.4 (SAS Institute Inc., Cary, NC, USA) was utilized for conducting all statistical analyses, and a significance level of 0.05 (two-tailed) was set.

## Results

We successfully implemented the computer assisted TFU protocol in the ED. For the analysis, we included 395 patients with TFU and 191 patients without TFU (Table 1). The median age in the TFU cohort and non-TFU cohort was 76 years and 74 years, respectively. The proportion of male sex in the TFU cohort and non-TFU cohort was 48.9% and 43.5%, respectively. Additionally, the TFU cohort showed a higher prevalence of Foley indwelling compared to the non-TFU cohort (8.6% vs. 3.7%, *p* = 0.028). Furthermore, the TFU cohort experienced a shorter LOS in the ED compared to the non-TFU cohort (median: 141 h vs. 188 h, *p* = 0.030). However, there was no significant difference in underlying comorbidities, including dementia, between the two cohorts. The comparison of LOS in the ED showed no significant difference in age subgroups between the two cohorts (Supplementary Table 1).

**Table 1:** Comparison of demographic characteristics and underlying comorbidities between TFU and non-TFU cohorts in older patients

Variable	TFU	Non-TFU	*p*-value
	*n* = 395	*n* = 191	
**Age (year), median (Q1 − Q3)**	76 (70 − 82)	74 (70 − 80)	0.065*
**Age subgroup, n (%)**			0.529
65 − 74 years	181 (45.8)	96 (50.3)	
75 − 84 years	140 (35.4)	65 (34.0)	
≥ 85 years	74 (18.7)	30 (15.7)	
**Sex, n (%)**			0.219
Female	202 (51.1)	108 (56.5)	
Male	193 (48.9)	83 (43.5)	
**Nasogastric tube, n (%)**	8 (2.0)	1 (0.5)	0.284^#^
**Foley indwelling, n (%)**	34 (8.6)	7 (3.7)	0.028
**Triage, n (%)**			0.871^#^
1	2 (0.5)	0 (0.0)	
2	67 (17.0)	30 (15.7)	
3	315 (79.8)	157 (82.2)	
4 + 5	11 (2.8)	4 (2.1)	
**Do not resuscitate, n (%)**	33 (8.4)	10 (5.2)	0.175
**LOS in ED (hour), median (Q1 − Q3)**	141 (92 − 262)	188 (107 − 282)	0.030*
**Underlying comorbidity, n (%)**			
Bedridden	119 (30.1)	54 (28.3)	0.645
Coronary artery disease	26 (6.6)	6 (3.1)	0.086
Congestive heart failure	53 (13.4)	24 (12.6)	0.775
**PAOD**	31 (7.9)	23 (12.0)	0.100
Cerebrovascular disease	119 (30.1)	56 (29.3)	0.841
**Dementia**	68 (17.2)	23 (12.0)	0.105
**COPD**	114 (28.9)	68 (35.6)	0.098
Rheumatic disease	26 (6.6)	12 (6.3)	0.890
Peptic ulcer disease	185 (46.8)	100 (52.4)	0.210
Liver disease	78 (19.8)	41 (21.5)	0.628
Diabetes	108 (27.3)	50 (26.2)	0.766
**Renal disease**	92 (23.29)	40 (20.94)	0.524
Malignancy	107 (27.1)	50 (26.2)	0.816

*Mann-Whitney U Test; ^#^Fisher’s Exact Test. Abbreviations: TFU, telephone follow-up; ED, emergency department; LOS, length of stay; PAOD, peripheral arterial occlusive disease; COPD, chronic obstructive pulmonary disease

In the univariate analysis, there were no differences in ED revisit < 3 days, ED revisit < 1 month, and mortality < 1 month between the two cohorts (Table 2). The number of hospitalizations < 14 days was higher in the TFU cohort compared to the non-TFU cohort (12.2% vs. 6.8%, *p* = 0.047). Additionally, the TFU cohort had a higher number of hospitalizations < 1 month compared to the non-TFU cohort (mean ± standard deviation: 0.2 ± 0.4 vs. 0.1 ± 0.3, *p* = 0.003). Moreover, the total medical expenditure was lower in the TFU cohort than in the non-TFU cohort (median: 4,467 New Taiwan Dollars [NTD] vs. 6,839 NTD, *p* < 0.001).

**Table 2:** Comparison of ED visits, hospitalizations, mortality, and total medical expenditure between TFU and non-TFU cohorts

Variable	TFU	Non-TFU	*p*-value
	*n* = 395	*n* = 191	
**ED revisit < 3 days, n (%)**	20 (5.1)	10 (5.2)	0.929
**ED revisit < 1 month**			
Total number, mean ± SD	0.2 ± 0.5	0.1 ± 0.4	0.138**
Subgroup of number, n (%)			
0	335 (84.8)	172 (90.1)	0.106^#^
1	44 (11.14)	14 (7.33)	
2	16 (4.1)	4 (2.1)	
3	0 (0.0)	1 (0.5)	
**Hospitalization < 14 days, n (%)**	48 (12.2)	13 (6.8)	0.047
**Hospitalization < 1 month**			
Total number, mean ± SD	0.2 ± 0.4	0.1 ± 0.3	0.003**
Subgroup of number, n (%)			
0	329 (83.3)	177 (92.7)	0.006
1	58 (14.7)	11 (5.8)	
2	8 (2.0)	3 (1.6)	
**Mortality < 1 month, n (%)**	11 (2.8)	3 (1.6)	0.565^#^
**Total medical expenditure < 1 month**			
Median (Q1 − Q3)	4,467 (2336 − 10,607)	6,839 (4343 − 10,690)	< 0.001*
Subgroup of total medical expenditure, n (%)			< 0.001
< 5,000 NTD	220 (55.7)	59 (30.9)	
5,000 − 15,000 NTD	95 (24.1)	100 (52.4)	
> 15,000 NTD	80 (20.3)	32 (16.8)	

*Mann-Whitney U Test; **T-test; ^#^Fisher’s Exact Test. Abbreviations: ED, emergency department; TFU, telephone follow-up; SD, standard deviation; NTD, New Taiwan dollars

The satisfaction of the index ED visit was higher in the TFU cohort than in the non-TFU cohort (mean ± standard deviation: 4.3 ± 0.6 vs. 3.9 ± 0.5, *p* < 0.001) (Table 3). Specifically, the satisfaction levels in the TFU cohort were as follows: 4.3 ± 0.5 for TCN, 4.1 ± 0.5 for TFU, 4.3 ± 0.6 for problem-solving, 4.1 ± 0.6 for transitional care, and 4.2 ± 0.5 for health education.

**Table 3:** Comparison of satisfaction of the index ED visit and TFU between TFU and non-TFU cohorts

Variable	TFU	Non-TFU	*p*-value
	*n* = 395	*n* = 191	
Satisfaction† of the index ED visit, mean ± SD	4.3 ± 0.6	3.9 ± 0.5	< 0.001*
Satisfaction† of the index ED visit, n (%)			< 0.001^#^
1	0 (0.0)	0 (0.0)	
2	5 (1.3)	1 (0.5)	
3	15 (3.8)	27 (14.1)	
4	243 (61.5)	152 (79.6)	
5	132 (33.4)	11 (5.8)	
Satisfaction of TCN, mean ± SD†	4.3 ± 0.5	−	
Satisfaction of TFU, mean ± SD†	4.1 ± 0.5	−	
Satisfaction of solving problem, mean ± SD†	4.3 ± 0.6	−	
Satisfaction of transitional care, mean ± SD†	4.1 ± 0.6	−	
Satisfaction of health education, mean ± SD†	4.2 ± 0.5	−	

*Mann-Whitney Test; ^#^Fisher; †Using five-points Likert Scale. Abbreviations: ED, emergency department; TCN, transitional care nurse; TFU, telephone follow-up; SD, standard deviation

Compared with the non-TFU cohort, the TFU cohort had a higher proportion of hospitalization < 1 month (AOR: 2.50; 95% CI: 1.31 − 4.77), lower total medical expenditure < 1 month (AOR: 0.32; 95% CI: 0.21 − 0.47), and higher satisfaction with the index ED visit (AOR: 2.80; 95% CI: 1.46 − 5.36) after conducting multivariate logistic regression analyses. The regression analyses included adjustments for age, sex, nasogastric tube, Foley indwelling, triage, do not resuscitate, LOS in the ED, underlying comorbidities, and ED visit time (Table 4).

**Table 4:** Comparison of outcomes, total medical expenditure, and satisfaction between TFU and non-TFU cohorts using multivariate logistic regression analyses

TFU vs. non-TFU (reference)	AOR (95% CI)*	*p*-value	AOR (95% CI)^#^	*p*-value
ED revisit < 3 days	0.95 (0.43 − 2.08)	0.896	0.92 (0.39 − 2.18)	0.849
ED revisit < 1 month	1.59 (0.92 − 2.75)	0.100	1.68 (0.92 − 3.05)	0.090
Hospitalization < 14 days	1.90 (1.00 − 3.60)	0.051	1.85 (0.93 − 3.68)	0.078
Hospitalization < 1 month	2.51 (1.37 − 4.60)	0.003	2.50 (1.31 − 4.77)	0.005
Mortality < 1 month	1.63 (0.45 − 5.96)	0.459	3.53 (0.24 − 52.44)	0.340
Total medical expenditure < 1 month (> 5,000 NTD)	0.36 (0.25 − 0.52)	< 0.001	0.32 (0.21 − 0.47)	< 0.001
Satisfaction of the index ED visit (Five-points Likert Scale > 3)	3.18 (1.74 − 5.84)	< 0.001	2.80 (1.46 − 5.36)	0.002

*Adjusted for age and sex; ^#^Adjusted for age, sex, nasogastric tube, Foley indwelling, triage, do not resuscitate, LOS in ED, bedridden, coronary artery disease, congestive heart failure, PAOD, cerebrovascular disease, dementia, COPD, rheumatic disease, peptic ulcer disease, liver disease, diabetes, renal disease, malignancy, and ED visit time. Abbreviations: ED, emergency department; TFU, telephone follow-up; AOR, adjusted odds ratio; CI, confidence interval; NTD, New Taiwan Dollars; LOS, length of stay; PAOD, peripheral arterial occlusive disease; COPD, chronic obstructive pulmonary disease

## Discussion

This study demonstrated the successful implementation of a novel computer assisted TFU. Compared with the non-TFU cohort, the TFU cohort had a lower total medical expenditure < 1 month, higher satisfaction with the index ED visit, but a higher proportion of hospitalization < 1 month.

Computer-assisted referral is a simpler and faster method compared to the conventional approach (i.e., contacting TCN physically or via telephone) as it is available 24/7 for referrals. The case management system also enhances the efficiency of TCN, allowing her to save more time while providing better patient care. This method has been proven to be effective in our previous implementation of home healthcare referrals after ED discharge [9]. The concept of computer-based referral for clinical practice was proposed as early as 1999 [17]. Sittig et al. developed a computer-based outpatient clinical referral application that facilitates the identification of an appropriate specialist, collection of necessary data for generating a referral, and transfer of information between the specialist and the primary care physician [17]. Their findings indicated that the new computer-based referral process is faster than conventional methods [17]. In our current study, the referral was carried out by ED physicians, or TCN for high-risk complications after ED discharge. Looking ahead, artificial intelligence-assisted identification with subsequent referral may represent an even more efficient method [18].

The reduced medical expenditure found in this study is a significant issue for patients, families, public health, and national health insurance, particularly in rapidly aging countries like Taiwan. There are few studies in the literature regarding the impact of TFU on medical expenditure. In Australia, a program named “Further Enabling Care at Home” implemented telephone intervention for caregivers of older people discharged from the hospital [19]. In that study, they utilized a specially trained nurse, like the TCN in our study, to address the support needs of caregivers of older patients discharged from the hospital [19]. They found that the medical expenditure (e.g., ambulance use, ED visit, or hospitalization) was $9,306 ± $13,734 in patients receiving telephone intervention and $9,421 ± $14,566 in the control group (*p* = 0.485) [19]. Similarly, a randomized control trial in the United States explored the effect of TFU among older adults discharged home from the ED and reported an estimated 70% chance that their intervention would reduce total costs [6]. The variation in medical services across different study countries may help explain the controversy.

Satisfaction is a patient-reported outcome that reflects the importance and quality of medical care, but it is not always measured in studies of medical services. Our study found that the TFU cohort had higher satisfaction than the non-TFU cohort, which contrasts with the results of previous studies. For instance, a pragmatic randomized controlled trial in older patients (≥ 70 years) discharged from ED in the Netherlands reported no satisfaction difference between the TFU and non-TFU groups [20]. In that study, they involved fifty-seven ED nurses and nine medical and nursing students for the TFU [20], which is different from our study, where we had only one dedicated TCN with ten years of experience in ED care. Although all ED nurses and medical and nursing students in their study were trained, there may be discrepancies compared to the dedicated TCN approach adopted in our study.

Our study revealed a higher hospitalization rate in the TFU cohort compared to the non-TFU cohort, which aligns with findings from previous studies. In a study conducted in the Netherlands, the unplanned 30-day hospitalization and/or ED return visit was observed in 16% of intervention group patients and 14% of control group patients (odds ratio 1.16; 95% CI: 0.96–1.42) [20]. Similarly, another study in the United States reported a hospitalization rate < 1 month of 9.0% in the TFU cohort, compared to 7.4% in the non-TFU cohort (not statistically significant) [7]. The possible reason for our result is that TFU may strengthen the awareness of disease deterioration in patients/caregivers, leading to a higher likelihood of patients/caregivers seeking emergency care due to the convenient, unlimited, and cost-effective nature of national health insurance in Taiwan. The higher hospitalization rate observed in the TFU cohort does not necessarily imply a positive outcome, as the causes of hospitalization were not investigated in our study. Further research on this issue is required. Another possibility is that the patients in the TFU cohort in our study were at higher risk for complications, either referred by the ED physicians or selected by the TCN. This higher risk is evident in the differences observed in age distribution, Foley indwelling, triage, and do not resuscitate status between the two cohorts.

This study demonstrated that the TFU cohort experienced a shorter LOS in the ED compared to the non-TFU cohort. A probable explanation for this difference is that the TFU cohort included patients with high-risk complications post-ED discharge, such as those discharged against medical advice, who might inherently have shorter ED stays. We did not collect detailed data on this aspect, as our primary aim was to compare outcomes between the TFU and non-TFU cohorts. Further investigation may be necessary to elucidate this issue.

Older people may encounter difficulties with new technology, including using telephones, due to hearing loss or blindness [21]. In this study, all patients receiving TFU had caregivers we could contact. However, it is important to consider those who do not have caregivers. Thus, designing e-health interventions that are accessible and user-friendly for older adults should account for their physical and sensory capabilities [21]. Developing solutions, such as voice-activated systems, screen readers, or tailored apps that meet the specific needs of users with sensory impairments, could mitigate these barriers and enhance the accessibility of telehealth services for older populations [21].

This study has several major strengths, including the successful implementation of a computer assisted TFU model in Taiwan, which can serve as a valuable reference for other hospitals. Furthermore, the study examines both health-related and patient-reported outcomes, providing a comprehensive evaluation of the intervention’s effects. Lastly, the study team comprises interdisciplinary healthcare professionals, ensuring a comprehensive evaluation of the intervention’s effectiveness. Overall, these strengths highlight the potential of this study to inform and improve emergency medical care for older patients. However, the study also has several limitations that should be considered when interpreting the findings. Firstly, the small sample size of the study and the lack of a one-to-one case comparison may not fully capture the actual differences between the TFU and non-TFU cohorts. To address this limitation, our hospital is currently conducting further studies with a larger sample size to improve the robustness of the results. Secondly, the ED TFU model implemented in this study may not be directly applicable to other hospitals or countries due to differences in medical resources and insurance systems. Modifications to the model may be necessary for other hospitals seeking to adopt it. Future studies in diverse settings and populations would be beneficial to validate the findings and assess the model’s effectiveness in different healthcare environments.

## Conclusions

This study demonstrated the successful implementation of a novel computer assisted TFU model, executed by a dedicated TCN. The 24/7 referral and case management system streamlined the referral and follow-up process, reducing paperwork for the TCN. The interdisciplinary team in the geriatric ED of the study hospital, with experienced geriatric care, provided strong support for the TFU model. Compared with the non-TFU cohort, the TFU cohort exhibited lower total medical expenditure < 1 month, higher satisfaction with the index ED visit, but a higher proportion of hospitalization < 1 month. These results are inconsistent with previous studies. We suggest that different protocols, medical expenditure, cultural factors, and insurance systems in various studies and countries may contribute to the observed discrepancies. However, our study’s findings are promising and offer an important reference for improving transitional care in older ED patients. Further research with a larger number of patients and validation in other hospitals are needed to strengthen the evidence and generalize the findings to a broader context.

### Electronic supplementary material

Below is the link to the electronic supplementary material.

Supplementary Material 1

## Data Availability

The datasets used and/or analyzed during the current study are available from the corresponding author upon reasonable request.
