# Supplementary material for: Implementation of a novel computer assisted telephone follow-up model for older patients after emergency department discharge in an Asian population
Source: Aging Clin Exp Res. 2024 Jul 18;36(1):147. doi: 10.1007/s40520-024-02796-6 (PMC11258097; doi:10.1007/s40520-024-02796-6)
Supplement: Supplementary file 1 — Supplementary Material 1 [file 40520_2024_2796_MOESM1_ESM.docx]

**Supplementary Table 1.** The LOS of ED in the patients classified by age subgroups.

|  | LOS in ED (hour), mean±SD | | *p*-value | LOS in ED (hour), median (Q1-Q3) | | *p-*value |
| --- | --- | --- | --- | --- | --- | --- |
| Age group | TFU | Non-TFU |  | TFU | Non-TFU |  |
| 65−74 | 216.1±248.4 | 239.7±284.0 | 0.494 | 134 (89−233) | 171 (97.5−258.5) | 0.206 |
| 75−84 | 218.5±238.5 | 323.4±698.7 | 0.243 | 150.5 (90−268.5) | 210 (122−294) | 0.402 |
| ≥85 | 272.5±337.1 | 215.3±136.6 | 0.221 | 151 (110−297) | 160.5 (113−287) | 0.731 |

Abbreviations: LOS, length of stay; ED, emergency department; SD, standard deviation; TFU, telephone follow-up.
